# Towards seasonal forecasting of malaria in India

**DOI:** 10.1186/1475-2875-13-310

**Published:** 2014-08-10

**Authors:** Jonathan M Lauderdale, Cyril Caminade, Andrew E Heath, Anne E Jones, David A MacLeod, Krushna C Gouda, Upadhyayula Suryanarayana Murty, Prashant Goswami, Srinivasa R Mutheneni, Andrew P Morse

**Affiliations:** Department of Earth, Atmospheric and Planetary Science, Massachusetts Institute of Technology, Cambridge, MA 02139 USA; School of Environmental Sciences, University of Liverpool, Liverpool, L69 7ZT UK; Institute of Infection and Global Health, University of Liverpool, Liverpool, L69 7BE UK; Atmospheric, Oceanic and Planetary Physics, Department of Physics, University of Oxford, Oxford, OX1 3PU UK; Council of Scientific and Industrial Research (CSIR) Fourth Paradigm Institute, Bangalore, 560037 India; Council of Scientific and Industrial Research (CSIR) Indian Institute of Chemical Technology (IICT), Hyderabad, 500607 India; National Institute of Health Research, Health Protection Research Unit in Emerging and Zoonotic Infections, Liverpool, L69 7BE UK

**Keywords:** Malaria, India, Seasonal forecasting, Disease modelling, Relative operating characteristic

## Abstract

**Background:**

Malaria presents public health challenge despite extensive intervention campaigns. A 30-year hindcast of the climatic suitability for malaria transmission in India is presented, using meteorological variables from a state of the art seasonal forecast model to drive a process-based, dynamic disease model.

**Methods:**

The spatial distribution and seasonal cycles of temperature and precipitation from the forecast model are compared to three observationally-based meteorological datasets. These time series are then used to drive the disease model, producing a simulated forecast of malaria and three synthetic malaria time series that are qualitatively compared to contemporary and pre-intervention malaria estimates. The area under the Relative Operator Characteristic (ROC) curve is calculated as a quantitative metric of forecast skill, comparing the forecast to the meteorologically-driven synthetic malaria time series.

**Results and discussion:**

The forecast shows probabilistic skill in predicting the spatial distribution of *Plasmodium falciparum* incidence when compared to the simulated meteorologically-driven malaria time series, particularly where modelled incidence shows high seasonal and interannual variability such as in Orissa, West Bengal, and Jharkhand (North-east India), and Gujarat, Rajastan, Madhya Pradesh and Maharashtra (North-west India). Focusing on these two regions, the malaria forecast is able to distinguish between years of “high”, “above average” and “low” malaria incidence in the peak malaria transmission seasons, with more than 70% sensitivity and a statistically significant area under the ROC curve. These results are encouraging given that the three month forecast lead time used is well in excess of the target for early warning systems adopted by the World Health Organization. This approach could form the basis of an operational system to identify the probability of regional malaria epidemics, allowing advanced and targeted allocation of resources for combatting malaria in India.

## Background

Malaria, a mosquito-borne infectious disease caused by parasitic protozoans of the *Plasmodium* genus, has a highly detrimental socio-economic impact on affected countries, presenting a significant public health challenge. Globally, in 2012, an estimated 3.4 billion people in 99 countries were at risk of contracting malaria with approximately 207 million reported cases and an estimated 627,000 reported deaths [1].

India is the most populous country affected by malaria, representing over 400 million people threatened by infection [2, 3]. Yet despite an extensive intervention campaign [4, 5] that resulted in near-eradication of malaria in India in the 1960’s, there are still between 1.6–15 million cases of malaria and between 1000–15,000 deaths reported per year [6, 7]. Indeed, a wide discrepancy has emerged between these figures, from primary health care facilities and the World Health Organization (WHO), and the actual burden of malaria in India [3, 6, 7], with one estimate suggesting an order of magnitude higher mortality rates of around 200,000 deaths per year [8]. This disparity may be associated with under-reporting of malaria fatalities, such as in rural regions where no health care professional may have been involved or in the private sector, and misdiagnosis of febrile symptoms [6, 8].

Temperature and rainfall influence the life cycle of female *Anopheles* mosquito vector and therefore the viability of the *Plasmodium* parasite within, vector breeding site availability and parasite transmission via the rate of human biting by the vector [9–15]. Epidemic outbreaks can occur as a result of climate anomalies, such as prolonged periods of rainfall (except rainfall extremes where flushing of mosquito larva becomes important) and in regions where malaria transmission is strongly seasonal such as semi-arid or highland regions [16–19]. Indeed, future climate changes could lead to an increase in malaria transmission in the marginally-suitable highland areas of Africa, South America and southeast Asia [20–24], although decreased precipitation in some regions may potentially reduce transmission [22]. In some regions, elevated temperature may lead to a reduction in mosquito survival and therefore, potentially reduced malaria transmission [25]. However, it is important into take account of continuing socio-economic development and human intervention measures [10, 21, 22, 26], which may have outpaced climate changes in the twentieth century resulting in reduced malaria extent and endemicity since the 1900’s [27]. Moreover, some marginal regions where malaria resurgence has recently occurred may well be more parsimoniously explained by reductions in vector control measures, increasing drug resistance, land use changes [28] and population growth rather than limited climate anomalies [29, 30], although this is debated [31, 32].

The WHO’s Roll Back Malaria global strategic plan suggests a target for malaria epidemic early warning systems to detect 60% of outbreaks within two weeks [33]. In India, an operationally useful early warning system combining meteorological data (rainfall) and human factors (nutritional vulnerability and “spleen infection index”) existed before the early 1950’s eradication programme [34, 35]. Progress has been made in India exploiting the relationships between climate and malaria to create dynamic disease models that are forced with observed or reanalysis time series of meteorological variables such as temperature, rainfall and humidity [36, 37]. However, attempts to actually predict malaria epidemics beyond short synoptic weather timescales have focussed on integrated or indirect methods such as surface vegetation cover as an indicator of water supply [38], monsoon signatures in South Atlantic sea-surface temperature [39] and correlations with the El Niño Southern Oscillation (ENSO) [40].

Using seasonal forecasts of meteorological variables could allow prediction of epidemic risk with potential lead times on the order of months. Following this methodology, seasonal hindcasts of meteorological variables have been successfully integrated with both prototype statistical-empirical and dynamic disease models to provide skillful hindcasts of malaria in Africa [12–15, 41, 42]. Predictive information such as this could then be used by health planners to target resources and logistics more effectively [7, 43].

In this study, a 30-year hindcast of malaria incidence in India is presented, produced by forcing a dynamic disease model with output from a state of the art coupled atmosphere-ocean seasonal forecast model (see the Methods section for details). In the first part of the Results section, the individual meteorological fields from the hindcast that are used to drive the disease model are verified against the corresponding fields from three meteorological observational/reanalysis data products (again, see the Methods section for details). This is also known as “Tier 1” validation [12]. It would then be preferable to validate the simulated malaria hindcasts against observational clinical records of malaria in India (“Tier 3” validation [12]). However, given that there has been extensive and continuing intervention in some regions of India (not captured by the disease model), that epidemiological records are inhomogeneous in space and time (which would hinder complete assessment of the malaria forecast) and that the observational data may not represent the true burden of malaria in India [3, 6–8], which might indicate lack of skill where it actually exists in the forecast, only a limited qualitative comparison with observational malaria data was possible. Only the spatial distribution of annual mean hindcast malaria transmission is compared against two aggregated time-slice estimates of contemporary (2010) and pre-intervention (approximately the year 1900) malaria (see Methods for further details). Another confounding factor for direct comparison between the malaria forecast and clinical malaria data is the necessity to downscale the forecast that has a spatial resolution on the order of 100’s km to the local scale on which the diagnosing facilities exist with minimal degradation of any forecast skill [12].

In lieu of a quantitative evaluation of the skill of the malaria forecast compared to clinical observations of malaria, a probabilistic assessment is carried out by comparing seasonal-averaged malaria incidence from the forecast to simulated malaria incidence synthesized by forcing the disease model with gridded meteorological observational fields. This is the well established intermediate technique of “Tier 2” verification [12, 13, 15]. Finally, the main results, perspectives and caveats of this study are summarized in the Discussion.

## Methods

### Seasonal forecasting system

The seasonal hindcast is based on simulations performed with a state of the art operational coupled ocean-atmosphere seasonal forecast model (“System-4”) developed at the European Centre for Medium-Range Weather Forecasts (ECMWF) [44]. The atmospheric component is the ECMWF Integrated Forecast System model (cycle 36r4) with a spatial resolution of approximately 0.7° ×0.7° and 91 vertical levels, with the top of the atmosphere at 0.01 hPa or 75 km. The System-4 ocean component uses version 3 of the Nucleus for European Modelling of the Ocean (NEMO) model [45] at approximately 1° ×1° resolution with refinement at the equator and 42 vertical levels (18 of which are in the upper 200 m). Initial conditions for the ocean are generated by the NEMOVAR ocean data assimilation system, while the atmosphere is initialized from an atmospheric reanalysis (the Interim ECMWF Reanalysis, see below).

**Figure 1:**
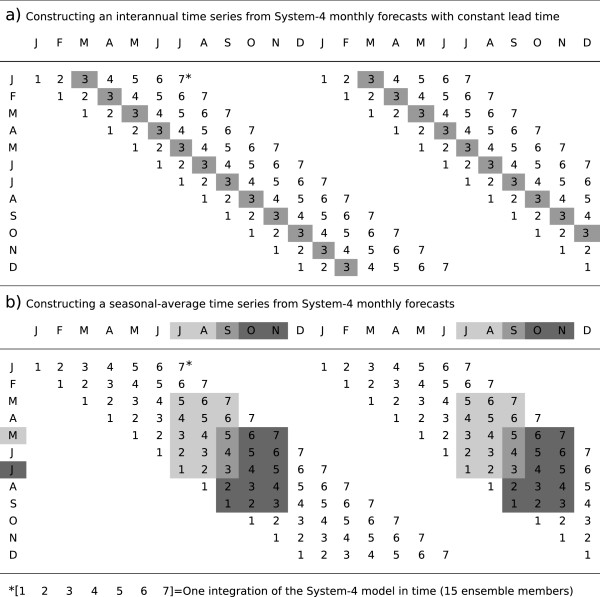
**System-4 forecast data selection schematic.** Twelve System-4 forecasts are issued for each year starting on the first of each month, extending for seven months per forecast. **a)** A single composite “monthly” time series was created by extracting a particular month from each forecast, resulting in a time series with constant forecast lead time (shaded months for a three month forecast lead time). **b)** For seasonal averages (the “seasonal” time series), the continuous forecast issued at the start of a particular month every year is used. The target seasons (shaded) are extracted leading to five potential seasonal averages per year with different forecast lead times. For a forecast lead time of three months, the JAS (SON) season is the average of months 3–5 from the forecast issued in each year in May (July).

Starting on the first of each month, 12 System-4 forecasts are issued in each past year, with a 15-member ensemble created using perturbations of the atmosphere and ocean initial state. From each start date, the 15 ensemble member forecast is run for seven months using a 45 minute time step in the atmosphere and a 3 hour ocean-atmosphere coupling interval. Daily rainfall and temperature from the System-4 hindcasts for India have been extracted for the period 1981–2010 within the domain 7–35°N and 66.5–97.5°E.A single “monthly” time series of System-4 meteorological drivers and simulated malaria incidence between 1981–2010 was created to allow comparison with the three observational data sets detailed below by extracting and then averaging daily output for a particular month for each of the 12 forecasts issued per year and each of the 15 ensemble members, resulting in an ensemble time series with constant forecast lead time (see the schematic in Figure 1a). For the later probabilistic analysis, a “seasonal” time series is extracted using the continuous forecast issued at the start of a particular month every year, with the daily data for a target season extracted and averaged according to a given forecast lead time (again, see the schematic in Figure 1b).

A three month forecast lead time was chosen in order to maximize forecast skill while decreasing the effect of persistence of the initial conditions of the forecast and the spin up of the disease model (see “The Liverpool Malaria Model” section below). For example, to investigate skill between September and November with a three month lead time, System-4 forecasts issued every July between 1981 and 2010 would be used, averaging over months three, four and five for the SON target season (Figure 1b). The reasoning behind this forecast lead time is threefold: (1) analysis of the five possible instances of each seasonal average indicates that in general, forecast skill decreases as a function of increasing lead time, the third month of a seven month forecast retains much of the skill in the simulated meteorological variables; (2) vector (and therefore parasite sporogonic development) lags behind seasonal precipitation, in which case precipitation from the preceding two to three months is likely to have a significant impact on peak malaria transmission [12, 13, 19]. The three month lead time is long enough to capture the preceding peak in monsoon rainfall as part of the forecast simulation, rather than influence the forecast with known monsoon rains as an initial condition if starting at a lead time of one or even two months; and (3) three month forewarning of climate suitability for a malaria epidemic would be a major boon to health planners for effective targeting of resources and logistics.

### Meteorological datasets

In order to verify the Indian System-4 hindcast, three observationally-based datasets are used. Contemporary daily temperature and rainfall time series are obtained from the Interim ECMWF Reanalysis (ERAI) [46], India Meteorological Department (IMD) gridded observations [47–51] and the Tropical Rainfall Measuring Mission (TRMM) Multi-satellite Precipitation Analysis (TMPA) [52].

ERAI is the latest global atmospheric reanalysis produced by ECMWF covering the period since 1979 with a spatial resolution of 1.5° ×1.5°. Reanalyses provide a spatially complete and coherent record of the evolution of the global atmospheric circulation, representing a “best guess” of the atmospheric state at a particular time using meteorological observations assimilated into a numerical model. In many regards, reanalyses are sometimes considered equivalent to observations. The model is essential to extrapolate the locally observed parameters to unobserved parameters or regions with few observations in a physically meaningful and consistent manner. In ERAI’s data assimilation cycle, all available observations are combined with prior information from the atmospheric model (i.e. the final state of the previous cycle) and a cost function is minimized consistent with errors associated with the input observations and the model parameterizations, using the 4D-Var method [46]. This analysis is used to initialize a short 12 hour integration the model, which provides the prior input for the next assimilation analysis and the cycle is repeated. ERAI data is available covering the entire System-4 hindcast period between 1981–2010.

The IMD datasets are gridded products based on station measurements interpolated onto a 1° ×1° regular grid covering India. For surface air temperature, 395 stations with quality-controlled daily data, which have at least 300 daily records per year for at least 10 years were used [47] covering the period 1969–2005. For rainfall, 1803 rain gauge stations were utilized based on the constraint that they have at least 90% daily data availability during the 50-year period of 1951–2003 [48, 49]. The selected time period of both temperature and rainfall datasets is from the start of System-4 hindcast availability, 1981, to 2002.

In addition, recently derived fine-scale rainfall observations from the TRMM Microwave Imager are utilized [53]. Although temporal coverage is limited to the last decade of the System-4 hindcast period (1998–2010), spatial resolution is significantly higher than either ERAI or IMD at 0.25° ×0.25°. The TRMM observations combine passive microwave, infrared and radar data from a constellation of satellite-borne precipitation-related sensors and *in situ* rain-gauge products between 50°S–50°N. The TRMM dataset has been shown to be consistent in the pattern and phase of intraseasonal variability of the Indian monsoon in other datasets [51, 54] and is largely independent of the other observations used here since passive microwave observations are excluded from the ERAI data assimilation pool [46], while the IMD rainfall dataset used here is not merged with TRMM dataset (not to be confused with the product presented in [54]).

### The Liverpool Malaria Model

The disease/climate impact model used in this study is the Liverpool Malaria Model (LMM [55]), which employs a dynamic, process-based approach to simulate malaria incidence in the human population, driven by daily time-series of rainfall and temperature. LMM couples a malaria transmission model and a dynamic mosquito population growth model. The rate of development of the parasite within the mosquito (the sporogonic cycle) and the mosquito biting rate (the gonotrophic cycle) are directly proportional to the number of “degree days” above the relevant threshold experienced by the mosquito (16°C [56] and 9°C, respectively). The gonotrophic cycle takes approximately 37 degree days whereas the sporogonic cycle takes approximately 111 degree days. Adult mosquito mortality is further governed by a quadratic function of temperature, where survival probability peaks at 20°C [57]. The vector component for most dynamical malaria models, especially the adult survival mortality scheme, rely on studies for *Anopheles gambiae* in Africa [41]. These parameters are deployed in this study in the absence of specific data for the dominant vectors (*Anopheles stephensi* and *Anopheles culicifacies*) in India.

The availability of surface water for mosquito breeding sites is simply parameterized by setting the quantity of eggs laid by each female mosquito to be proportional to the previous ten days’ (dekadal) rainfall as a proxy for a more complex hydrological model incorporating land-surface heterogeneity. Larval mosquito mortality rate is also dependent on dekadal rainfall. Further details of the model formulation are available elsewhere [12–15, 55].

This configuration relates specifically to a single malarial parasite, *Plasmodium falciparum*. Compared to the other *Plasmodium* (such as *Plasmodium malariae*, *Plasmodium ovale* and *Plasmodium vivax*), *P. falciparum* leads to the most severe symptoms such as multiple organ failure and constitutes approximately half of total modern malaria cases in India [4, 58]. A significant contribution to the burden of malaria in India comes as a result of infection by *P. vivax* [28, 43], however this is not considered here.

Malaria forecasts were produced at every grid point, for each of the 30 years of the System-4 time series from 1981 to 2010, for each of the 12 monthly forecast start dates and for each of the 15 model ensemble members. The LMM was initialized with the correct preceding year’s reanalysis data (from ERAI) for a spin-up period of one year prior to the forecast start date. The use of ERAI data for initialization of the disease model required the System-4 hindcast variables to be interpolated onto the 1.5° ×1.5° grid and therefore the malaria hindcast is produced at a slightly coarser resolution.

Three baseline references for “Tier 2” malaria forecast validation were created by running LMM with the entire daily time series of temperature and precipitation for the reanalysis data (ERAI) and the gridded observations (IMD). The fine-scale satellite (TRMM) data, which only provides rainfall, was coupled with an interpolated ERAI temperature time series to drive the disease model. Therefore differences between the overlapping ERAI and hybrid “TRMM-ERAI” malaria time series can be largely attributed to differences in rainfall intensity and distribution.

None of the hindcast data were bias corrected to allow the skill of the meteorological inputs to be mapped to the skill of the impact model outputs. Indeed, it has been documented that in some cases bias correction may actually reduce forecast skill [13].

### Observations of malaria in India

Despite the aforementioned caveats regarding malaria observations and how they relate to the true burden of malaria in India, in order to attempt some external validation the annually-averaged output of the malaria model, the hindcast and observational datasets were compared to published annually-averaged malaria prevalence maps of *P. falciparum* in 2010 from the Malaria Atlas Project (MAP2010) [2, 59]. The MAP2010 dataset is a statistical model constrained by survey data from nearly 13,500 administrative units in 85 countries totalling over 22,000 unique data points. However, the observed survey data is relatively sparse over India with respect to western and eastern Africa. These data points were combined with environmental remote-sensing data and socio-economic predictors in a Bayesian model to produce a best guess spatial distribution of malaria transmission on a 5 ×5 km grid. We have interpolated the data to the regular grid of 0.25° ×0.25°, which matches our finest resolution meteorological observational data (TRMM).

Pre-intervention malaria endemicity estimates [60] were also compared to the simulated distribution of malaria in India. These estimates (digitized from the original paper and rasterized onto a 5 ×5 km grid [27, 61]) are based on a major synthesis of historical records, documents and maps of a variety of malariometric indices performed in 1968 for the four major *Plasmodium* species for the nominal year 1900 based on a wide variety of indices (disease records, vector presence and absence, spleen rates, parasite rates, sickle cell incidence, sporozoite rates, biting rates, expert opinion and climate variables) classified into categories of parasite rate in the 2–10 age group as hypoendemic, mesoendemic and hyperendemic. The holoendemic class is determined by parasite rate in the one-year-old age group alone. This is the most reliable compilation for the historical peak in malaria and is reported to compare well with contemporary local maps of malaria incidence [27].

The relationship between malaria and climate is more likely to manifest in the 1900’s distribution of malaria transmission as the disease was not inhibited by significant control or treatment measures. Nevertheless, other socio-economical factors not captured by LMM, such as worker migration and agricultural irrigation [28], may also have influenced the malaria distribution at that time causing differences between model and observations.

### Relative Operating Characteristic (ROC) skill scores and probabilistic analysis

Beyond qualitatively comparing the climatological spatial distribution and average seasonal cycle of malaria between the different simulations, a quantitative measure of skill can be evaluated by performing a Relative Operating Characteristic (ROC) analysis [62–64].

ROC curves were originally designed for use in signal detection of objects using radar during World War II. Given a series of instances (where there is or is not an object on the radar) and a classifier who can interpret a signal as representing an object or just noise (no object) then these binary pairs can be divided into a “confusion matrix”, or “contingency table”, of the number of events that were correctly classified (true positives, or “hits”), objects that were classified as noise (false negatives, or “misses”), noise in the signal that was interpreted as an object (false positives, or “false alarms”) and noise that was correctly classified as noise (true negatives, or “correct rejections”). ROC curves portray the compromise between the true positive identification rate (0 to 1 on the y-axis) and false positive identification rate (0 to 1 on the x-axis), with each set of instances representing a discrete point in ROC space.

A classifier could randomly guess hits in half of the instances and will correctly identify half of the time but also misclassify half the time. This behaviour lies in ROC space at the central point (0.5, 0.5). Similarly, a classifier that randomly guesses hits a quarter of the time plots at (0.25, 0.25) in ROC space while a classifier that randomly guesses hits three-quarters of the time will plot at (0.75, 0.75). A classifier that never issues a hit will never register a true positive, but will also issue no false alarms and so plots in the lower left at A(0, 0). Conservative classifiers cluster in near this corner and require strong evidence before issuing a hit. Conversely, a classifier that unconditionally issues hits will have a perfect true positive identification rate, but will also score a maximum false alarm rate, thus plotting on the upper right at B(1, 1). Liberal classifiers that cluster near this corner issue hits with weak evidence. These pre-determined classification strategies form a diagonal between AB in ROC space. In order to perform better than random and plot above AB, the classifier must draw on evidence in the signal to inform the decision to issue a hit. A perfect classifier correctly identifies all objects and noise (with no false alarms) and plots in the upper left of ROC space at (0, 1). Finally, it is possible to perform worse than random and plot below the random diagonal, AB. Usually when this occurs an error has been made by the classifier misinterpreting useful information in the signal. By inverting all the classifier’s decisions, a point above AB can be recovered. ROC analysis has been extended for use in psychology, machine learning and medical decision making.

For the Tier 2 comparison, malaria output from the disease model driven by the three meteorological observation/reanalysis products and the System-4 hindcast ensemble are classified into three separate “events” in which malaria transmission in a particular season was low (below the lower tercile), above average (above the median) and high (above the upper tercile). The event horizons were computed from the spread of simulated malaria incidence values for each simulation of LMM driven by the different forcing datasets, therefore the frequency of certain event classifications rather than the specific magnitude of the events in each instance is important. The 15 ensemble members of the System-4 hindcast allows the probability of a particular event occurring to be calculated, where the ensemble members were equally-weighted when calculating forecast probabilities.

To construct a ROC curve the meteorological-data-driven LMM malaria time series is used to determine if an “event” occurred or not (this is the essence of a Tier 2 analysis, in a Tier 3 analysis event occurrence would be determined from clinical observations) and then the System-4 forecast probabilities are used as the classifier, employing a range of threshold probabilities to decide if a hit is issued. Each threshold produces a single point in ROC space, therefore a range of thresholds from high probability to low probability is used for the curve. “Averaging” of ROC curves is performed to display skill in forecasting the interannual variability of malaria in different subregions. This is achieved by collating the individual test sets (that is a test instance for each grid point over the duration of the data) into a single overarching dataset on which the ROC analysis is performed [62].

The ROC plot can be summarized by calculating the area under the curve, which always lies between 0 and 1 and is not usually less than 0.5 due to the area under the diagonal created using a random guessing strategy of classification. This metric provides the probability that the classifier will rank a randomly chosen positive instance higher than a randomly chosen negative instance [62]. Significance levels for the ROC area value are calculated by a comparison to a critical value of the two-tailed Mann-Whitney U-statistic [65] where the significance level, *p*, is 0.05.

## Results

In the following, apart from the pan-Indian analysis, two key regions are focussed on: the states of Orissa, West Bengal, and Jharkhand (NE India between 20–27°N and 83–88°E), and the states of Gujarat, Rajastan, western Madhya Pradesh and western Maharashtra (NW India between 16–25°N and 68–78°E) as they are areas where high modern malaria transmission is reported a could be susceptible to climate-driven malaria epidemics [6, 7, 19, 43, 58].

### Meteorological drivers

**Figure 2:**
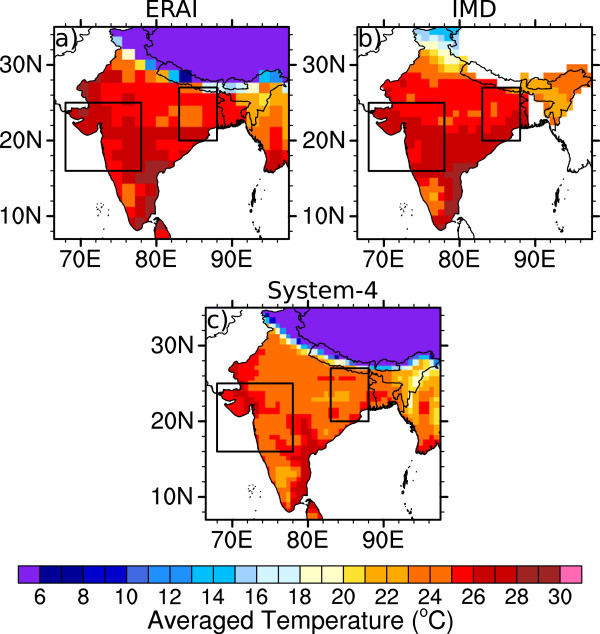
**Annual average 2 m surface air temperature (°C).** Values are shown for **a)** ERAI (1981–2010), **b)** IMD (1981–2002) and **c)** the System-4 forecast (1981–2010) monthly time series with a three month lead time (see Figure 1a). The two boxes enclose the regions of interest in Northwest and Northeast India.

The distribution and magnitude of annually-averaged surface temperatures between ERAI, IMD and the System-4 forecast monthly time series (with a lead time of three months, see Figure 1a) are broadly similar (Figure 2), with the highest temperatures in a band from the Gulf of Khambhat in the Northwest, across central India to the region of Chennai city in southwest. The System-4 forecast monthly time series also captures the cool regions on the southeast coast (Kerala and west Tamil Nadu), the far eastern “Seven Sisters” states of India (Assam and Arunachal Pradesh among others), Bangladesh and Myanmar, and the high-altitude regions of Jammu and Kashmir, Nepal and China compared to ERAI and IMD. Although the temperature distribution is similar to the observational datasets, the System-4 temperatures are somewhat cooler than the observations by approximately one to two degrees. However, in the IMD dataset, there is a large temperature bias of nearly 5°C in the disputed Jammu and Kashmir region (the Northern-most Indian state) where only a limited number of station measurements were available to produce the gridded data [47].

**Figure 3:**
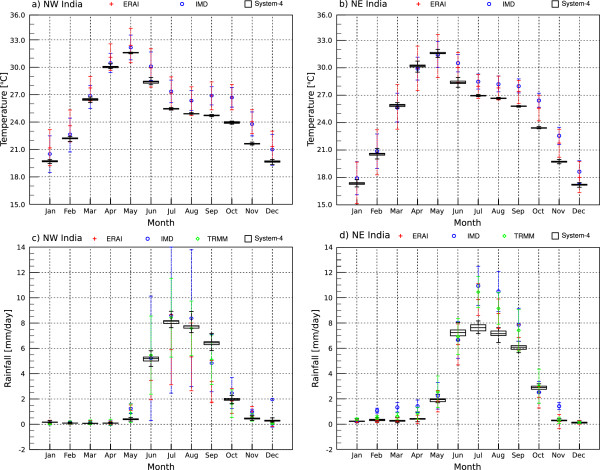
**Average seasonal cycles of temperature (°C) and precipitation (mm/day).** Values are shown for ERAI (1981–2010), IMD (1981–2002), TRMM (1998–2010) and the System-4 forecast (1981–2010) monthly time series (see Figure 1a) with a three month lead time for **a)** temperature in Northwest India, **b)** temperature in Northeast India, **c)** rainfall in Northwest India and **d)** rainfall in Northeast India. Meteorological data spatial variability is illustrated by plus or minus one standard deviation of the mean. The System-4 values are plotted as a box that shows the upper tercile, lower tercile and the mean of the 15 equally-weighted ensemble forecast members and the whiskers indicate the maximum and minimum values.

In both the NW and NE India regions, the seasonal cycle of temperature (Figure 3a and 3b) peaks in May in all datasets during the summer, with slightly cooler temperatures during the monsoon season between June and September before temperatures drop to below 20°C between November-January. Again, the cooler forecast bias compared to the observational datasets is clear, particularly in the latter part of the year.

**Figure 4:**
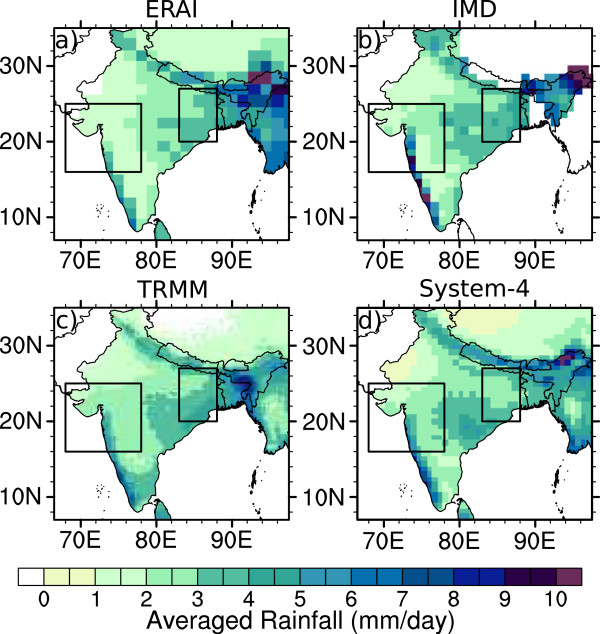
**Annual average precipitation (mm/day).** Values are shown for **a)** ERAI (1981–2010), **b)** IMD (1981–2002), **c)** TRMM (1998–2010) and **d)** the System-4 forecast (1981–2010) monthly time series with a three month lead time (see Figure 1a). The two boxes enclose the regions of interest in Northwest and Northeast India.

Annually-averaged rainfall also compares well between the System-4 forecast monthly time series and the ERAI, IMD and TRMM datasets (Figure 4). There is a band of high rainfall along the length of the west coast (Kerala to Gujarat), a drier area in central regions (inland Karnataka, Andhra Pradesh, Maharashtra and Madhya Pradesh) and a return to wet conditions at 20°N from the east coast that extends inland to the west of 80°E (Orissa, Chhattisgarh and coastal Andhra Pradesh). Only the TRMM satellite data suggests elevated precipitation in Tamil Nadu in southern India, while again the IMD data for rainfall in the Jammu and Kashmir region of Northern India is inconsistent with the other datasets probably as a result of insufficient measurements available to produce the gridded dataset [48, 49].

The seasonal cycle of rainfall (Figure 3c and 3d) shows mostly dry conditions between November and April and wetter conditions during the monsoon season between June to September, peaking in July. The monsoon rains tend to begin slightly earlier during May in the NE India region compared to June in the NW India region [66, 67]. The spread between the different datasets and the ensemble spread within the System-4 forecasts during the monsoon season is much more evident than for temperature as a result of the geographical variation of the different rainfall patches in Figure 4. For example, ERAI is relatively dry in NW India compared to the other datasets because of a weaker central Indian rainfall band extending west along 20°N from the Bay of Bengal. Similarly, in NE India, the System-4 forecasts are relatively drier than the other datasets due to slightly less rainfall over the Ganges Plain. Although the magnitudes vary between datasets, the timing of the start and end of the rainy season is successfully captured, even by the three-month System-4 forecast monthly time series.

Overall, the System-4 climatological distribution and seasonal cycle of temperature and rainfall compares well to the three observed datasets.

### Malaria transmission

**Figure 5:**
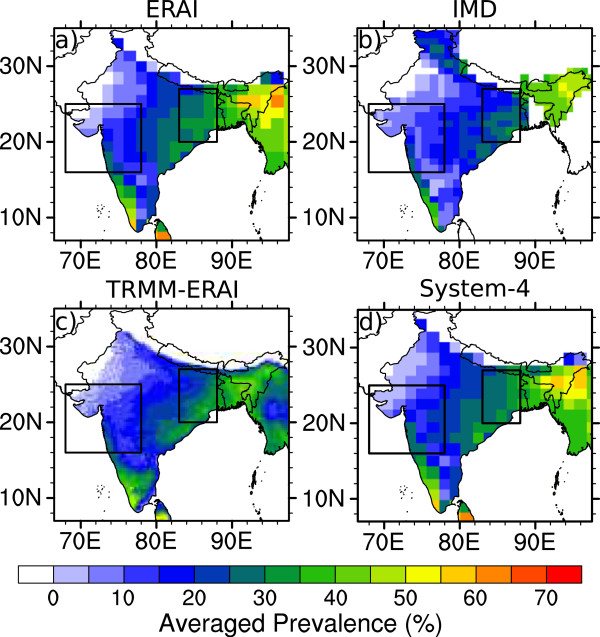
**Annual average malaria prevalence (%).** Output is shown from the Liverpool Malaria Model (LMM) driven by **a)** ERAI (1981–2010), **b)** IMD (1981–2002), **c)** TRMM precipitation and ERAI temperature (TRMM-ERAI, 1998–2010) and **d)** the System-4 forecast (1981–2010) monthly time series with a three month lead time (see Figure 1a). The two boxes enclose the regions of interest in Northwest and Northeast India.

Integrating the temperature and precipitation data from the observational, reanalysis and forecast drivers through LMM results in four time series of simulated malaria incidence. The annually-averaged spatial distribution of malaria in India is shown in Figure 5 with a three month lead time for System-4 forecast monthly time series. These data were all interpolated onto the same 1.5° ×1.5° grid. The climate is suitable for malaria transmission along the southwest coasts of Kerala, Karnataka, Goa and Maharashtra, the Northeastern region of India in West Bengal, Jharkhand, Orissa and Bihar as well as Arunachal Pradesh, Assam, Meghalaya, Manipur, Mizoram, Nagaland and Tripura in the east. Insufficient station data in the meteorological drivers in the Jumma and Kashmir region of the IMD gridded data translate into relatively high simulated malaria transmission in upland regions, where the other datasets and forecasts agree that the climate is unsuitable.

**Figure 6:**
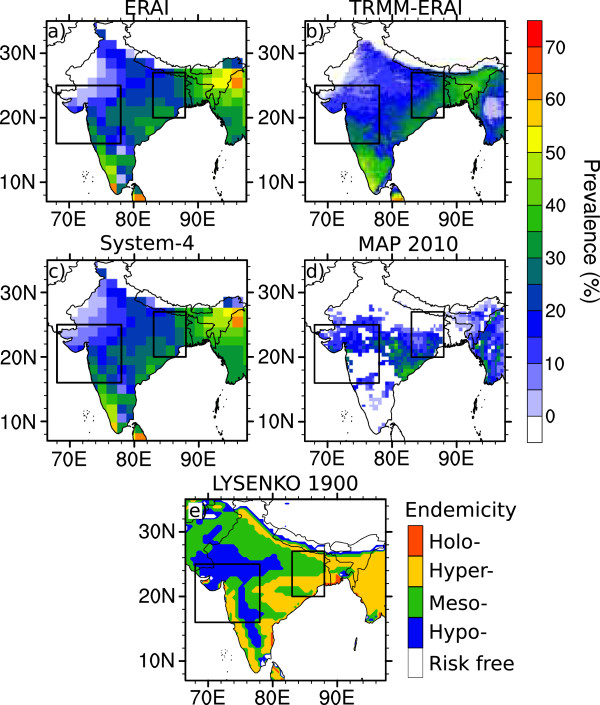
**Annual average malaria prevalence for 2010 only (%).** Output is shown from the Liverpool Malaria Model (LMM) for the year 2010 driven by **a)** ERAI, **b)** TRMM precipitation and ERAI temperature (TRMM-ERAI) and **c)** the System-4 forecast monthly time series with a three month lead time (see Figure 1a). Note that the IMD time series extends only to 2002 and is therefore absent in this comparison. Two observationally-derived Indian malaria estimates are also provided for qualitative Tier 3 comparison with the disease model: **d)** the prevalence of *P. falciparum* from the Malaria Atlas Project (MAP 2010) [59] and **e)** a pre-intervention (circa 1900) malaria distribution [60, 61]. The two boxes enclose the regions of interest in Northwest and Northeast India.

As mentioned previously, there would be only limited gain from a comprehensive comparison between malaria distribution simulated by LMM and clinical observations from India due to factors that are not captured by the disease model such as extensive intervention in some regions and potential underreporting of the true burden of malaria. However, these simulations can be qualitatively compared to the distribution of *P. falciparum* occurrence (prevalence estimates in the 2-10 year old population) obtained by the Malaria Atlas Project (MAP) for the year 2010 [59] (Figure 6d). Simulated malaria for each data source has been extracted and averaged for the same year (Figure 6a-c) except for the IMD-driven run, which only has data until 2002.

Compared to the MAP2010 malaria prevalence, the simulated malaria transmission displays a largely consistent pattern with elevated levels in coastal Andhra Pradesh, southern Orissa and eastern Madhya Pradesh. However, LMM misses the moderate malaria rates observed in Gujarat (although TRMM-ERAI does feature elevated malaria rates there) and overestimates malaria transmission in western Rajasthan. The impact of climate on malaria may be limited in areas where there is intensive industrial and agricultural activity covering the Gujarat and Maharashtra regions where more socio-economic and land-use factors such as irrigation and worker migration are more important for malaria transmission [4, 16–18, 28, 38]. High malaria simulated over the western coast of India (Kerala to Gujarat) from LMM does not fit the observed distribution either as there have been extensive malaria control programmes in these areas (for example in Goa [5]). Furthermore, prevalence in MAP2010 is significantly lower than model estimates over the eastern states such as Assam and Arunachal Pradesh.

Simulated climatic suitability for malaria transmission from LMM is more reminiscent of the pre-intervention endemicity estimates of the parasite rate in children under 10 for the year 1900 (Figure 6e) [27, 60, 61], although again only a qualitative comparison is possible (see both Figure 5 and Figure 6). There is extensive malaria transmission along the west coast states of Kerala, Karnataka, Goa and Maharashtra, the Northeastern region of India in the states of Orissa, Jharkhand and Chhattisgarh and in the eastern “Seven Sisters” states. Lower values are found along the border with Pakistan and in the interior of the Indian peninsula, which is largely consistent in all four LMM integrations, especially the System-4 forecast monthly time series with a three-month lead time. Furthermore, this distribution again suggests that the climate is unsuitable for malaria in the Jumma and Kashmir region in Northern India. Nevertheless, malaria transmission in Gujarat and western Rajasthan appears to still be underestimated by LMM compared to the pre-intervention malaria distribution, again highlighting socioeconomic causes or malaria model biases such as not taking account of multiple parasite species [43]. These patterns are quite similar to those obtained with other malaria models such as “Modelling framework for the health impact assessment of man-induced atmospheric changes” (MIASMA), based on monthly rainfall and temperature [68].

**Figure 7:**
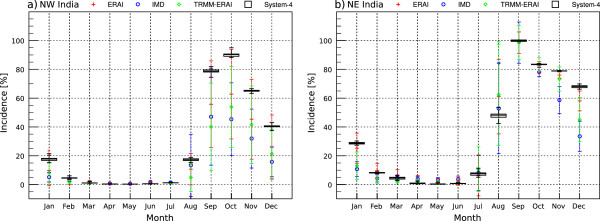
**Average seasonal cycle of malaria incidence (%).** Values are shown for ERAI (1981–2010), IMD (1981–2002), TRMM precipitation and ERAI temperature (TRMM-ERAI, 1998–2010) and the System-4 forecast monthly time series with a three month lead time (see Figure 1a) in **a)** Northwest India and **b)** Northeast India. Meteorological data-driven incidence spatial variability is illustrated by plus or minus one standard deviation of the mean. System-4 values are plotted as a box that shows the upper tercile, lower tercile and the mean of the 15 equally-weighted ensemble forecast members and the whiskers indicate the maximum and minimum values.

Malaria incidence in both NW India and NE India has a distinct seasonality (Figure 7), with little or no malaria between February and June and elevated incidence following roughly two months after the start of the rainfall season. In NE India, the monsoon rains appear in MJJ [66, 67] producing increasing malaria risk in JAS whereas in NW India, where the monsoon begins slightly later in JJA [66, 67] with malaria risk elevated during SON. The System-4 monthly malaria time series performs well compared to the malaria time series simulated from meteorological observations/reanalysis data, although it is generally at the upper range of the malaria time series and particularly overestimates malaria incidence in NW India. In this region, the high rainfall and lower temperatures in System-4 forecasts, while still above the sporogonic and gonotrophic thresholds, could act to prolong the lifespan of the simulated mosquitoes, therefore elevating malaria transmission risk. The rainfall-temperature-mosquito survival interplay appears to cancel in the NE India region, where System-4 rainfall is initially lower than in the observational datasets, however mosquito survival is slightly enhanced compared to the other datasets due to the System-4 cool temperature bias.

None of the System-4 hindcast data in this study were corrected to account for long term differences in input temperature or precipitation from climatology. It would have been possible to adjust the average climate of the System-4 hindcast by bias correction, for example by considering the long-term departure from monthly anomalies or separately correcting the frequency and intensity of weather events [13, 15] or statistical bias correction methods such as statistical downscaling, quintile mapping, histogram equalization/matching [69] and adjustment using mapped empirical orthogonal functions derived from the hindcast and applied to the forecast [70]. These adjustments could account for a proportion of climate bias in the Indian subregions, for example due to shifted precipitation patterns. However, extreme care is required due to the non-linear combination of input variables integrated by the disease model. Indeed, it is useful to determine the skill of the malaria hindcast from the raw input data since meteorological comparison data for a forecast does not exist and therefore simple bias correction methods risk introducing greater error [13]. Recall that event horizons for the skill analysis below were computed from the spread of simulated malaria incidence values for each simulation of LMM driven by different forcing datasets, therefore the frequency of certain event classifications rather than the specific magnitude of the events in each instance is important.

The LMM integrations show quite large year to year variability in malaria incidence in simulated low or moderate transmission areas such as in Gujarat and central-western India (NW India) denoting a higher risk of epidemic malaria outbreaks over those regions [19]. In the probabilistic, event-based analysis that follows, this becomes important since LMM does not simulate immunity in the human population and therefore is more meaningful in areas at risk of epidemics. Nevertheless, the seasonal nature of malaria incidence for both the NW and NE India regions (Figure 7) initiated by monsoon rainfall also suggest vulnerability to malaria epidemics associated with anomalous climatic variability [16, 17].

**Figure 8:**
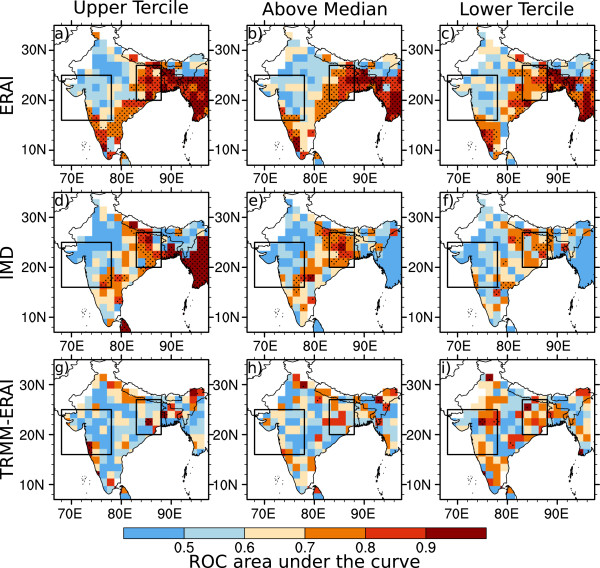
**Skill maps of System-4 malaria forecasts in July, August and September.** Skill maps of malaria incidence show the area under the ROC curve for the System-4 malaria forecast seasonal time series (see Figure 1b) averaging months three to five from a May start date against **a-c)** ERAI (1981–2010), **d-f)** IMD (1981–2002) and **g-i)** TRMM precipitation and ERAI temperature (TRMM-ERAI, 1998–2010) for high (above the upper tercile, left column), above average (above the median, central column) and low (below the lower tercile, right column) malaria occurrence. Regions where the ROC area is statistically-significant are stippled. The two boxes enclose the regions of interest in Northwest and Northeast India.

The distribution of values of area under the ROC curve for the System-4 seasonal time series issued in May for malaria transmission between July and September (see Figure 1b) compared to the three malaria time series simulated by the disease model driven by the three meteorological observation/reanalysis datasets (as in a Tier 2 analysis) are shown in Figure 8.

There are high ROC area values of greater than 0.7 in the NE India region for high malaria events (above the upper tercile), higher than average events (above the median) and low events (below the lower tercile). Indeed many of these events are significantly skillful at the 95% confidence level compared to critical values of ROC area from the two-tailed Mann-Whitney U-statistic [63] (stippled regions). The three-month lead time System-4 malaria seasonal time series in the NE India region is significantly skillful for high and above average malaria events compared to both the ERAI (Figure 8a-c) and IMD (Figure 8d-f) datasets whereas there does appear to be some skill but it is marginally significant for low malaria events and for all events when comparing System-4 forecasts to the TRMM-ERAI dataset (Figure 8g-i), probably because of the short length of the time series: only 13 years compared to 30 years for ERAI and IMD.

**Figure 9:**
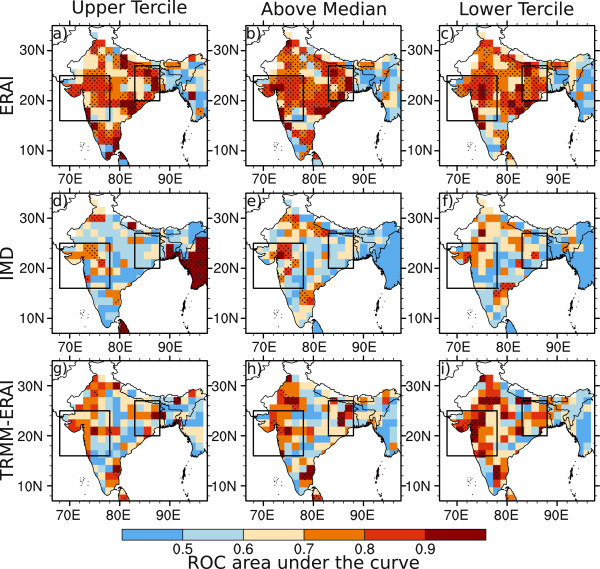
**Skill maps of System-4 malaria forecasts in September, October and November.** Skill maps of malaria incidence show the area under the ROC curve for the System-4 forecast seasonal time series (see Figure 1b) averaging months three to five from a July start date against **a-c)** ERAI (1981–2010), **d-f)** IMD (1981–2002) and **g-i)** TRMM precipitation and ERAI temperature (TRMM-ERAI, 1998–2010) for high (above the upper tercile, left column), above average (above the median, central column) and low (below the lower tercile, right column) malaria occurrence. Regions where the ROC area is statistically-significant are stippled. The two boxes enclose the regions of interest in Northwest and Northeast India.

For the System-4 seasonal time series issued in July for malaria transmission between September and November (see Figure 1b, again in a Tier 2 sense), there are high values of ROC area across central and North India compared to all three observed datasets (Figure 9). In particular, there is significant skill in this season for all events compared to ERAI across the entire country, where malaria transmission is already in progress in both the NE India and NW India regions. There is moderate skill in NW India for high and above average events in the System-4 forecast compared to the IMD malaria time series and some is significant, particularly in the Gujarat and Maharashtra regions, while only moderate skill is found for low malaria events. In the NW India area, there is somewhat significant skill in the malaria forecasts compared to the TRMM-ERAI malaria time series for above average and low events in particular, again limited by the short length of the time series.

The effect of persistence of initial conditions in LMM simulations has been previously investigated [13, 15] by creating a “control run” forecast ensemble where the malaria model was driven with the correct spin up data for a given year, followed by data for the forecast period taken from observational or reanalysis data for each remaining “wrong” year. For simulations in Africa with a lead time of four months (three month lead times were not tested), this persistence forecast was found to not be skillful, meaning any skill in the forecast was not from the initial conditions used during the LMM spin up process but the simulated variables during the forecast period. For this study a slightly different approach was taken, using long-term average reanalysis data instead of the correct year’s data during the year-long spin up of the disease model. Initially, for the target seasons with one to two month lead times, there is no skill in the System-4 malaria forecasts. However, there is significant skill when the target season has a three month forecast lead time, suggesting skill in System-4 malaria forecasts is indeed from the variables simulated during the forecast period and not from the initial conditions.

**Figure 10:**
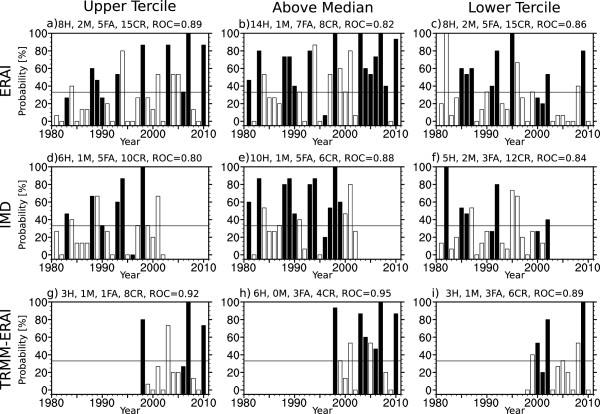
**Interannual skill time series of System-4 malaria forecasts in September, October and November for the North-western region of India.** Probability of a malaria “event” occurring in the NW box of the System-4 malaria forecast seasonal time series (see Figure 1b) averaging months three to five from a July start date against **a-c)** ERAI (1981–2010) temperature and precipitation, **d-f)** IMD (1981–2002) gridded temperature and precipitation observations and **g-i)** TRMM precipitation and ERAI temperature reanalysis (TRMM-ERAI, 1998–2010). The three events considered are high (above the upper tercile), above average (above the median) and low (below the lower tercile) malaria occurrence. See the text in the “Malaria transmission” section of the results for interpretation.

**Figure 11:**
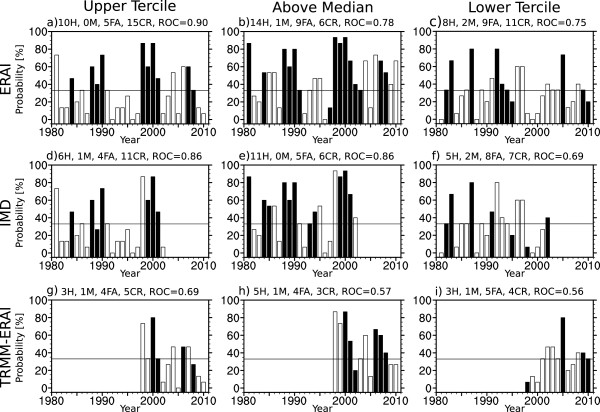
**Interannual probability time series of System-4 malaria forecasts in July, August and September for the North-eastern region of India.** Probability of a malaria “event” occurring in the NE box of the System-4 malaria forecast seasonal time series (see Figure 1b) averaging months three to five from a May start date against **a-c)** ERAI (1981–2010) temperature and precipitation, **d-f)** IMD (1981–2002) gridded temperature and precipitation observations and **g-i)** TRMM precipitation and ERAI temperature reanalysis (TRMM-ERAI, 1998–2010). The three events considered are high (above the upper tercile), above average (above the median) and low (below the lower tercile) malaria occurrence. See the text in the “Malaria transmission” section of the results for interpretation.

The performance of the malaria predictions can be viewed in time as well as space to demonstrate the skill of the System-4 forecast seasonal time series with three month lead time as a prototype early warning system for the last 30 years compared to the Tier 2 malaria time series generated from the observational or reanalysis data-driven LMM runs as a reference (Figure 10 and Figure 11). The bars are filled when the observationally-driven disease model runs (ERAI, IMD or TRMM-ERAI) indicate an event (malaria levels above the upper tercile, above the median and below the lower tercile malaria incidence), whereas non-events in the observationally-driven disease model runs are unfilled bars. It is perfectly acceptable for there to be no events indicated in any particular year, which suggests that malaria incidence lies between the lower tercile and the median. Similarly, a particular year may be associated with two events since all upper tercile events are also above median events, but not all above median events trigger and upper tercile event, suggesting malaria incidence lies between the median and the upper tercile. The event probabilities on the y-axis are calculated from the 15 System-4 malaria forecast ensemble members. Given a critical forecast event probability of 33% (an arbitrary, but relatively liberal value for the weight of evidence), a true positive result, or “hit” (H), is issued in a year where the observationally-driven disease model runs indicates an event and when the probability of an event in the System-4 malaria forecast exceeds the 33% threshold. In other words, *filled* bars that have a probability of greater or equal to 33% are considered hits. On the other hand, a false negative, or “miss” (M), is issued in a year where the observationally-driven disease model runs indicate an event but the probability of an event in the System-4 malaria forecast does not exceed the 33% threshold. In other words, *filled* bars that have a probability of less than 33% are considered misses. A false positive, or “false alarm” (FA), occurs in a year where the observationally-driven disease model runs do not indicate a malaria event but the System-4 probability of an event is above the 33% threshold (that is an *unfilled* bar with a System-4 probability of greater or equal to 33%). Finally, a true negative, or “correct rejection” (CR), of an event occurs when neither the observationally-driven disease model runs nor the System-4 malaria ensemble predicts an event (that is *unfilled* bars with a forecast probability or less than 33%). The ROC value for the area from the aggregated dataset is also indicated (see Methods). The results for the System-4 malaria forecast compared to the three meteorologically-driven simulated malaria time series for the three event classes in the two regions are summarized as confusion matrices in Table 1.

**Table 1:** **Confusion matrix for Tier 2 evaluation of the System-4 malaria forecast seasonal time series**

a.) JAS	Event forecast in System-4										
Event in ERAI (1981–2010)	>UT	Yes	No		>M	Yes	No		<LT	Yes	No
	Yes	10	0		Yes	14	1		Yes	8	2
	No	5	15		No	9	6		No	9	11
Event in IMD (1981–2002)	>UT	Yes	No		>M	Yes	No		<LT	Yes	No
	Yes	6	1		Yes	11	0		Yes	5	2
	No	4	11		No	5	6		No	8	7
Event in TRMM-ERAI (1998–2010)	>UT	Yes	No		>M	Yes	No		<LT	Yes	No
	Yes	3	1		Yes	5	1		Yes	3	1
	No	4	5		No	4	3		No	5	4
b.) SON	Event forecast in System-4										
Event in ERAI (1981–2010)	>UT	Yes	No		>M	Yes	No		<LT	Yes	No
	Yes	8	2		Yes	14	1		Yes	8	2
	No	5	15		No	7	8		No	5	15
Event in IMD (1981–2002)	>UT	Yes	No		>M	Yes	No		<LT	Yes	No
	Yes	6	1		Yes	10	1		Yes	5	2
	No	5	10		No	5	6		No	3	12
Event in TRMM-ERAI (1998–2010)	>UT	Yes	No		>M	Yes	No		<LT	Yes	No
	Yes	3	1		Yes	6	0		Yes	3	1
	No	1	8		No	3	4		No	3	6

The confusion matrix, or contingency table, collates hit, miss, false alarm and correct rejection rates in Figures 10 and 11 for the System-4 seasonal time series against the three meteorological observation/reanalysis datasets used to drive LMM for a.) the target season of July, August and September for the NE India region (forecasts issued in May) and b.) the target season of September, October and November for the NW India region (forecasts issued in July). The different event categories (read horizontally) are abbreviated as “ ***>***UT” for malaria incidence above the upper tercile, “ ***>***M” for malaria incidence above the median and “ ***<***LT” for malaria incidence below the lower tercile.

The System-4 malaria hindcast seasonal time series correctly classifies the majority of high, above average and low malaria events compared to ERAI, IMD and TRMM-ERAI time series in the NW India region for the SON malaria season and in the NE India region for the JAS malaria season, with a sensitivity (hit rate) of between 75–95% on average and a specificity (correct rejection rate) of around 75% for high malaria events and just over 50% for above average and low malaria events (see Table 2). The overall Tier 2 accuracy of the System-4 malaria forecast is 71% on average, even given the relatively short time series for the TRMM-ERAI hybrid dataset.

**Table 2:** **Statistics for Tier 2 performance of the System-4 malaria forecast seasonal time series**

		JAS (NE India)			SON (NW India)		
		ERAI	IMD	TRMM-ERAI	ERAI	IMD	TRMM-ERAI
		(1981–2010)	(1981–2002)	(1998–2010)	(1981–2010)	(1981–2002)	(1998–2010)
	Sensitivity	1.00	0.86	0.75	0.80	0.86	0.75
	Specificity	0.75	0.73	0.56	0.75	0.67	0.89
	Precision	0.67	0.60	0.43	0.62	0.55	0.75
	Negative predictive	1.00	0.92	0.83	0.88	0.91	0.89
Above upper tercile	value						
	Accuracy	0.83	0.77	0.62	0.77	0.73	0.85
	ROC area	0.90	0.86	0.69	0.89	0.80	0.92
	ROC critical value	0.73	0.77	0.86	0.73	0.77	0.86
	Sensitivity	0.93	1.00	0.83	0.93	0.91	1.00
	Specificity	0.40	0.55	0.43	0.53	0.55	0.57
	Precision	0.61	0.69	0.56	0.67	0.67	0.67
	Negative predictive	0.86	1.00	0.75	0.89	0.86	1.00
Above median	value						
	Accuracy	0.67	0.77	0.62	0.73	0.73	0.77
	ROC area	0.84	0.88	0.57	0.82	0.88	0.95
	ROC critical value	0.71	0.83	0.84	0.71	0.83	0.84
	Sensitivity	0.80	0.71	0.75	0.80	0.71	0.75
	Specificity	0.55	0.47	0.44	0.75	0.80	0.67
	Precision	0.47	0.38	0.38	0.62	0.63	0.50
	Negative predictive	0.85	0.78	0.80	0.88	0.86	0.86
Below lower tercile	value						
	Accuracy	0.63	0.55	0.54	0.77	0.77	0.69
	ROC area	0.75	0.89	0.86	0.88	0.84	0.89
	ROC critical value	0.73	0.77	0.86	0.73	0.77	0.86

The System-4 seasonal malaria time series is compared against the three meteorological observation/reanalysis datasets used to drive LMM. The three events considered are high malaria occurrence (above the upper tercile), above average malaria (above the median) and low malaria (below the lower tercile). Using values from the confusion matrices in Table 1, the sensitivity, or “hit rate”, is the number of hits divided by the total number of hits and misses, the specificity, or “correct rejection rate”, is the number of correct rejections divided by the total number of false alarms and correct rejections, the precision is the number of hits divided by the total number of hits and false alarms, the negative predictive value is the number of correct rejections divided by the total number of correct rejections and misses and the accuracy, which is the number of hits and correct rejections divided by the total number of events. Also shown are values of area under the ROC curve and it’s critical value for significance (where the significance level, *p*, is 0.05) calculated from the two-tailed Mann-Whitney U-statistic [63].

The ROC areas in both regions for their respective malaria seasons are generally above 0.75, reaching high skill levels of greater than 0.8, and are largely statistically significant compared to critical values computed from the two-tailed Mann-Whitney U-statistic [63], except for the short TRMM-ERAI dataset. The TRMM-ERAI also performs more poorly for malaria in NE India for above average events, where ROC areas drop to 0.57 and are noticeably lower than than compared to ERAI and IMD for high malaria events (0.69).

## Discussion

The Liverpool Malaria Model was used to produce a 30 year malaria hindcast for India between 1981–2010. Comparison of the meteorological variables used to drive the disease model from the forecast and three meteorological observation and reanalysis datasets revealed that the distribution of temperature and precipitation is accurately reproduced between the System-4 hindcast monthly time series with a three month lead time and ERAI, IMD and TRMM time series (Tier 1) with a cool bias in temperature and slightly wet bias in annually-averaged rainfall in NW India and a dry bias in NE India. The seasonal cycle was also successfully reproduced, capturing peak temperatures in May and the seasonal monsoon rains between June and September, with the rains starting slightly earlier in May in NE India compared to June in NW India [66, 67].

Although the System-4 malaria hindcasts have not been extensively verified against clinical data (Tier 3), limitations in these data associated with heterogeneity in space and time, the effect of human interventions and possible under-reporting of the true malaria burden in India [3, 6–8] limit the usefulness of such an exercise. Tier 3 comparison of the System-4 malaria hindcast to a suitable observational time series for India would be useful to assess the validity of using LMM parameters derived from studies in Africa, which were used in the absence of specific parameter values for India. Nevertheless, a qualitative comparison to the estimated distribution of annually-averaged *P. falciparum* prevalence in 2010 [59] indicates that the System-4 hindcast monthly time series reproduces the observed malaria hotspots over Assam and Arunachal Pradesh, over the industrial/mining belt of West Bengal, Jharkhand and Orissa, while it misses the Rajasthan and Gujarat malaria hot spots, as transmission here might be related to other socio-economic factors that are not captured by LMM. Climate is also highly suitable for malaria transmission over the western coast of India, but extensive control program have drastically reduced malaria transmission over these regions, thus hiding the climatic effect. A reconstructed “pre-intervention” malaria distribution [27, 60, 61] is highly reminiscent of the climate suitability of malaria transmission generated by LMM forced by the System-4 hindcasts and also by three separate integrations of LMM driven by the three observational meteorological datasets.

The System-4 malaria hindcast seasonal time series shows statistically significant skill in being able to predict the spatial distribution and interannual variability of malaria transmission in seasonal, epidemic-prone regions of India compared to malaria incidence simulated by the three observed meteorological datasets in an impact focussed Tier 2 evaluation. Individual years can be used as case studies for the System-4 malaria forecasts. For example, the 2009 monsoon was considered an anomalous drought year (that is, below one standard deviation of the seasonal-average rainfall) [54] and indeed, the System-4 forecasts for malaria compared to the ERAI and TRMM-ERAI datasets for the NW India region (Figure 10) predict a low malaria event and correctly reject high and above average events. In NE India, System-4 successfully forecasts low malaria events and correctly rejects high malaria events compared to ERAI and TRMM-ERAI datasets. Above average malaria transmission is correctly rejected compared to TRMM-ERAI but a false alarm is sounded by a few percent probability compared to ERAI (Figure 11). A reduction in malaria cases in the mid-80’s was observed after a period of stabilization following national malaria reemergence in the 1960’s [58] with an increased number of forecast low malaria events during this period particularly in NW India. Similarly, flood years (one standard deviation above the seasonal average rainfall) such as 1988 and 1994 [67, 71] are correctly forecast as above average or high malaria transmission years. Again, a national resurgence in malaria cases in the mid-90’s was indeed observed [58]. In 2007, a particularly strong monsoon, the System-4 malaria forecasts compared to ERAI and TRMM-ERAI show high malaria transmission in both regions. One of the advantages of using a forecast-disease model system as opposed to relying on pan-Indian climate indices such as monsoon intensity to anticipate malaria epidemics is illustrated by considering recent years. For example 2010 was correctly forecast as a high or above average malaria year compared to the ERAI and TRMM-ERAI time series in the NW India region, whereas in NE India malaria incidence is correctly forecast as low compared to TRMM-ERAI but just missed compared to ERAI alone. This spatial variability in the monsoon, even during a particular monsoon season, is not unexpected and can be associated with the passage of monsoon depressions and position of the monsoon trough over India [66]. It should be noted that operational System-4 forecasts have a significantly larger number of ensemble members, 51 compared to 15 available for hindcasts between 1981 and 2010 [44], and as such, skill from these operational forecasts is potentially higher than skill estimate using hindcast data.

Despite being based on relationships between components of the malaria life cycle that are relatively well known, if tuned somewhat for studies in Africa [41], the LMM malaria simulations are limited by several assumptions such as a single vector-parasite malaria complex, which reduces applicability of the forecasts where several forms of *Plasmodium* coexist, the lack of immunity acquisition by the human population and the inclusion of other socio-economic factors that influence malaria transmission rates. Inclusion of a simple parameterization of a treatment for *P. vivax* relapses in a dynamic disease model [43] demonstrated the efficacy of such a treatment in significantly reducing malaria cases.These factors should be addressed in the next generation of vector-borne disease/climate impact models [72]. Furthermore, the use of native relatively large grid resolution for seasonal forecasts and observations does not capture the heterogeneity of land surface cover that provides mosquito breeding sites, which is heavily parameterized in LMM, nor the highly patchy nature of actual malaria transmission by mosquitoes. Best practice for scaling down even relatively high resolution data such as the 25 km TRMM data to the scale of seasonal standing water pools requires further investigation [12].

Influence of the initial conditions on malaria forecast skill is also an important consideration. Previous studies using a specially constructed control, persistence forecast show no skill compared to meteorological data [13, 15], while using climatological average data for the LMM spin up do not show skill until three months into the forecast, indicating that the skill originates from the actual simulated meteorological variables produced during the forecast rather than the data used for initialization, particularly capturing the rainfall peak in the System-4 integration rather than seeding the disease model with observed rainfall for each year as would be the case for a shorter forecast lead time. Furthermore, potential persistence of the ERAI atmospheric state used to initialize the System-4 forecast at ECMWF is also minimized by choosing a significantly long forecast lead time. Of course, this is one of the reasons to use other meteorological data to force the disease model and compare to the System-4 malaria forecast and indeed significant skill is found, although the TRMM-ERAI observations are somewhat too short in duration. Furthermore, skill in ERAI could also be the result of the comprehensive set of meteorological observations that are assimilated, compared to the gridded station measurements of IMD and the microwave/rain gauge observations in TRMM. One particular source of skill in the System-4 malaria forecast could come from the use of a coupled atmosphere-ocean model: Tropical sea surface temperature exerts an important influence on the Indian monsoon [39], thus explicitly simulating changes in ocean state may allow for robust forecasting of precipitation as can be seen in the greater similarity in Figure 4 between the climatological distribution of rainfall from System-4 and direct observations from IMD and TRMM, compared to ERAI, which although assimilates rainfall data does not include a sophisticated ocean model.

Since the malaria hindcasts used here only provide information about the climatic suitability for malaria epidemics of *P. falciparum*, they should really only be treated as indicators of malaria risk in appropriate regions where socio-economic malaria factors are not considered important. Inclusion of *P. vivax* into the malaria complex would allow more robust malaria forecasts. Further validation at Tier 3 should be pursued by attempting to compare the System-4 malaria hindcast with clinical malaria incidence should a suitably long and clean record exist and the downscaling of the relatively coarse resolution System-4 forecast to local scales on which the clinical data exist be suitably accurate [12]. Nevertheless, these are encouraging results given that the three month lead time used here is well in excess of the target for early warning systems adopted by the WHO and could prove to be a useful part of the toolkit available to decision makers, providing benefits through advanced and targeted allocation of resources for combatting malaria epidemics in India.
